# Challenges and strategies in high-accuracy manufacturing of the world’s largest SiC aspheric mirror

**DOI:** 10.1038/s41377-022-00994-3

**Published:** 2022-10-26

**Authors:** Xuejun Zhang, Haixiang Hu, Xiaokun Wang, Xiao Luo, Ge Zhang, Wenxing Zhao, Xiaoyi Wang, Zhenyu Liu, Ling Xiong, Erhui Qi, Congcong Cui, Yanchao Wang, Yingjie Li, Xu Wang, Longxiang Li, Yang Bai, Qiang Cheng, Zhiyu Zhang, Ruigang Li, Wa Tang, Xuefeng Zeng, Weijie Deng, Feng Zhang

**Affiliations:** 1 Changchun Institute of Optics, Fine Mechanics and Physics, Chinese Academy of Sciences, 130033 Changchun, Jilin China; 2 University of Chinese Academy of Sciences, 100049 Beijing, China

**Keywords:** Astronomical optics, Optical metrology

## Abstract

In the process of manufacturing the world’s largest silicon carbide (SiC) aspheric mirror, the primary difficulties are mirror blank preparation, asphere fabrication, and testing, as well as cladding and coating. Specifically, the challenges include the homogeneity of the complicated structure casting, accuracy and efficiency of the fabrication process, print-through effect, fidelity and precision of test procedure, stress and denseness of cladding process, the dynamic range of interferometric measurement, and air turbulence error due to the long optical path. To break through such a barrier of difficulties, we proposed the water-soluble room temperature vanishing mold and gel casting technology, homogeneous microstructure reaction-formed joint technology, nano-accuracy efficient compound fabrication, gravity unloading technology, high-denseness low-defect physical vapor deposition (PVD) Si-cladding technology, test data fusion method, and time-domain averaging method, etc. Based on the proposed technologies and methods, we have accomplished the world’s largest SiC aspheric mirror with a size of ⌀4.03 m. The impressive performance of the SiC aspheric mirror is validated by the characteristics of the fabricated SiC aspheric mirror. The aerial density of the SiC blank is less than 120 kg/m^2^, surface shape test accuracy is better than 6 nm RMS, thickness inhomogeneity of the cladding layer is less than 5%, and the final surface figure error and roughness are 15.2 nm RMS and 0.8 nm RMS, respectively.

## Abstract (web summary)

CIOMP report their progress in high-accuracy manufacturing of the world’s largest SiC aspheric mirror. The final surface figure error is 15.2 nm RMS and aerial density is less than 120 kg/m^2^.

## Graphical abstract

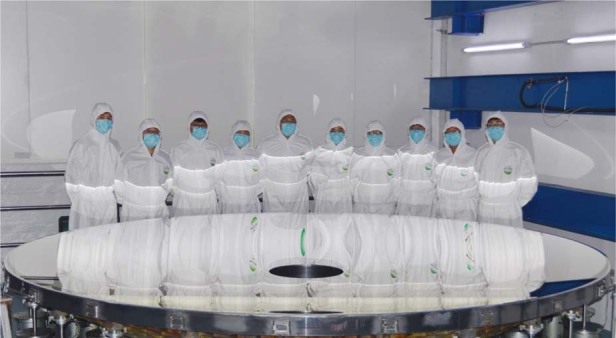

## Introduction

Aspherical components used in optical systems can increase the free design variables without introducing new aberrations, and that brings improved imaging quality and reduced size and weight. Therefore, aspheric components are widely used in high-end optoelectronic instruments, such as space and ground-based astronomical telescopes, deep space exploration and earth observation optics, deep/extreme ultraviolet (DUV/EUV) lithography optics, and high-performance photographic cameras^1–6^.

The two critical specifications of a telescope system are the angular resolution (AR) and light collecting capacity (LCC), which are closely related to the aperture of the system^7–11^. AR is inversely proportional to the diameter of the telescope while LCC is proportional to its squared value^12,13^. The larger the aperture, the higher and stronger the AR and LCC. Consequently, increasing the aperture becomes the essential way to improve the telescope’s performance, and that is the reason why both astronomy and earth observing communities need large telescopes.

However, the larger size of the primary mirror in modern ground-based telescopes and space cameras imposes demanding stringent requirements on mirror materials and full-spatial frequency (FSF) shape errors control^14,15^. Therefore, breakthroughs in mirror materials, accurate and efficient manufacturing of large aspherical mirrors are urgently expected^16^.

Compared to the counterpart mirror materials, such as ULE® and Zerodur®, silicon carbide (SiC) is of higher specific stiffness (SS) and dimensional stability (DS), and is well suited for harsh environment applications. Moreover, the reaction-bonded silicon carbide (RB-SiC) process can produce a semi-closed back structure which further increases its SS and DS. Therefore, a large aperture SiC mirror with superior mechanical and thermal properties has soon become a new favorite in the telescope community worldwide^17^. Nevertheless, manufacturing large SiC mirrors (i.e., diameter > ⌀2 m) faces extremely difficult challenges related to the preparation process of SiC mirror blanks, which is accompanied by complex physical phase changes and chemical reactions. Furthermore, the currently existing technology is not adapt to mirrors over ⌀1.5 m^18^; the difficulty of manufacturing aspheric mirrors is proportional to the third power of the aperture; the equipment and technology for polishing optical class SiC mirror larger than ⌀2.5 m is not commercially available. The largest magnetorheological finishing (MRF) polishing equipment is the QED Q22-2200f made in the United States, with a maximum processing size of 2.5 m^19^.

In 2014, French companies, Reosc and Boostec, reported the largest monolithic SiC mirror blank for a visible light application, which is a 1.54 × 0.49 m rectangular sintered SiC mirror. Its aspherical shape accuracy is 9 nm RMS^20^, and the accuracy-to-size ratio (ASR) is 5.84 ppb. ASR is defined as the ratio of the surface shape error RMS to the diameter of the optical component. Herschel telescope’s primary mirror used to be the largest SiC mirror (⌀3.5 m) ever made^21^. However, its shape accuracy is limited to 3 μm RMS due to the welding stress deformation of the brazing process. Consequently, the mirror is suited for far-infrared and submillimeter band applications.

Currently, the chemical vapor deposition (CVD) cladding process is an adopted cladding technique for overcoming the inherent surface defects of SiC^22^. However, the substrate in this case usually needs to be heated up to 1000 °C^23^, the large thermal shock may lead to irreversible mirror shape deformation. In addition, the cladding layer formed by ion source-assisted electron beam evaporation exhibits relatively poor denseness and adhesion. More importantly, the cladding process involves a considerable risk of cracking the SiC mirror.

In the last two decades, Changchun Institute of Optics, Fine Mechanics and Physics, Chinese Academy of Sciences (CIOMP, CAS) has investigated and developed a whole processing chain for manufacturing large optical class SiC mirrors. It covers mirror blank preparation by reaction-bonding process, aspherical surface computer numerical control (CNC) generating, computer-controlled optical surfacing (CCOS), Si cladding (or overcoating) on the substrate surface by means of physical vapor deposition (PVD) process, and high-accuracy asphere computer-generated hologram (CGH) interferometry, etc.

## Results

**Fig. 1:**
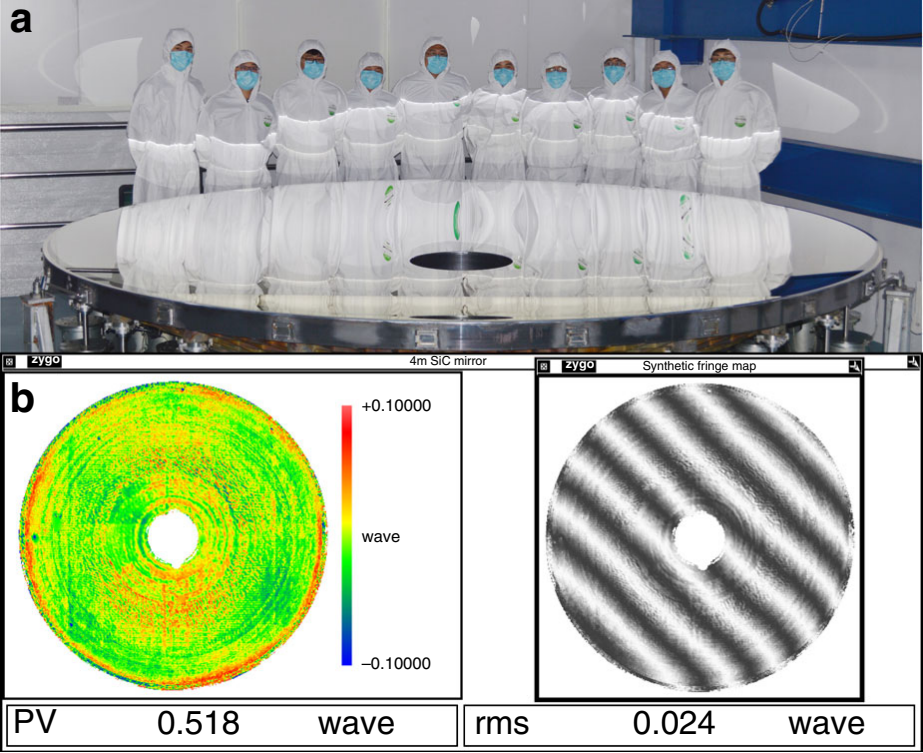
⌀4.03 m SiC aspheric mirror manufacturing results. **a** Picture of the world’s largest SiC aspheric mirror. **b** Test result and interferogram of the ⌀4 m SiC aspheric mirror

CIOMP has completed a ⌀3 m monolithic SiC mirror blank, and a ⌀4.03 m SiC mirror blank by reaction-formed joint technology. Using a home-built MRF^24^ polishing machine (maximum processing range is ⌀4.5 m), a ⌀4.03 m SiC aspheric mirror was polished to the shape accuracy of 15.2 nm RMS and ASR of 3.77 ppb, as shown in Fig. 1. As a result, the clear aperture of the mirror reaches ⌀4006 mm with a ⌀650 mm inner hole. Hence, the neglected edge effect is about 12 mm for each side. The mirror’s aerial density is less than 120 kg/m^2^, and the interferometric testing accuracy is better than 6 nm RMS, with RMS and PV uncertainties of ±0.2 and ±30 nm, respectively.

We proposed the PVD cladding process to improve the substrate surface quality. In this process, a 10–20 μm-thick Si-cladding layer was deposited on the substrate surface, and its defects (pores) were covered up by Si material. Thanks to the low processing temperature (<50 °C), very small deformation (<5%) occurred during the cladding process.

The ⌀4.03 m aspheric SiC mirror has been delivered to the customer in 2019 and operated well on site. Compared to the Herschel primary mirror, the delivered mirror is of a larger clear aperture (⌀4.03 m) and much higher shape accuracy (15.2 nm RMS).

## Discussion

SiC mirror manufacturing process is composed of lightweight mirror blank preparation, accurate and efficient asphere fabrication, precision measurement, Si overcoating, and self-weight deflection decoupling as well.

Current sintering process used to produce monolithic SiC mirrors less than ⌀2m^20^, the constrain are due to the high shrinkage ratio (>10%) and risk of light weighting process by CNC milling. The brazing technique was adopted to produce larger mirrors, such as Herschel’s primary, the welding stress-induced deformation limits its application to far-infrared and submillimeter band telescopes.

Based on the reaction-bonding technology, CIOMP came up with a set of material preparation techniques, such as the gel casting technology via water-soluble room temperature vanishing mold and crack-free drying, to generate a complex lightweight green body, SiC binder, and joining process to stitch segments into a monolithic blank. As a result, a ⌀4.03 m SiC aspheric mirror blank has been produced.

To overcome the low efficiency of the conventional fabrication process, we proposed a new processing chain, combining the CNC generating, stressed lap grinding/polishing, and CCOS and MRF polishing. As a result, the error convergence rate was increased by 30%, and FSF error control has been achieved. The low-temperature PVD Si cladding has proven to be a good way to cover up the substrate defects and improve the surface quality. The allocated cycle time for manufacturing the ⌀4.03 m SiC aspherical mirror is shown in Table 1.

**Table 1:** Allocated cycle time in manufacturing the ⌀4.03 m SiC aspheric mirror

Process	Generating	Grinding	Cladding	Polishing	Coating
Cycle Time (weeks)	30^a^	32^b^	14^c^	22	4

^a^Generating process includes optical surfacing phase and mechanical surfacing phase

^b^Grinding process includes loose abrasive grinding and coarse polishing on SiC substrate surface

^c^Cladding process includes mirror cleaning and Si layer overcoating

**Fig. 2:**
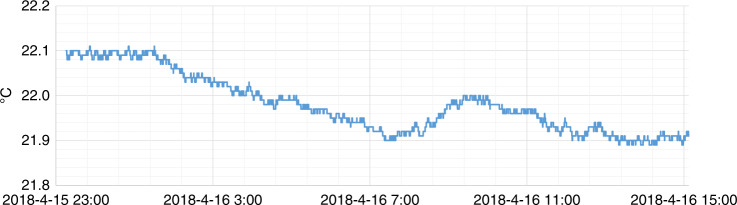
Temperature variations during the measurement over 12 h. Testing environment temperature is maintained at about 22 ℃ with no more than 0.2 ℃ fluctuation

To deterministically fabricate a ⌀4.03 m SiC aspheric mirror, a high-precision measurement technique is demanded. Since quantitative test datum are needed from generating (mm precision) to polishing (nm precision) to guide the CCOS process, there is no single test instrument having such a wide dynamic range. Based on the existing measurement techniques such as swing arm profilometry (SAP), CGH null interferometry, and phase deflectometry, we proposed a test data fusion technique, which seamlessly combines the test data from different sources. For the ⌀4.03 m SiC aspheric mirror, the measurement accuracy and reproducibility are 6 nm RMS and 2.6 nm RMS, respectively. The temperature range is set to 21.8–22.2 °C and maintained with a 0.2 °C precision over 12 h, as shown in Fig. 2. The RMS and PV variations are 15.2 ± 0.2 nm and 328 ± 30 nm, respectively, which is mainly due to signal noise, thermal instability, and air turbulence^25^. Meanwhile, the mirror self-weight deflection and air turbulence-induced error were decoupled by means of gravity unloading and temporal averaging technique.

## Materials and methods

### Large high structural rigidity SiC mirror blanks manufacturing technology

**Fig. 3:**
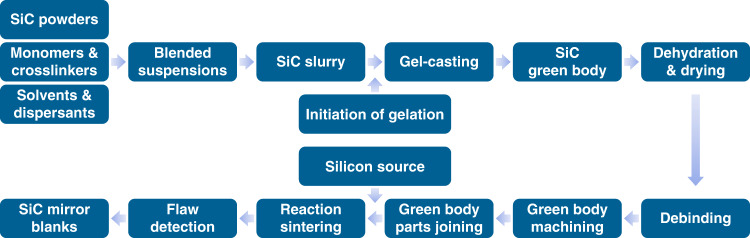
The preparation procedure of the ⌀4.03m SiC mirror blank. A flow chart from powders to mirror blanks

Figure 3 shows the CIOMP’s process chain to produce the ⌀4.03 m RB-SiC mirror blank. Unlike the conventional sintering process, the lightweight green body was formed by means of water-soluble room temperature vanishing mold and gel casting technology. Then, a crack-free liquid drying technique was adopted to ensure the near-net shape structure and low stress. During the vacuum reaction sintering, SiC binder and the joining process was used to stitch 12 segments into one monolithic blank^26–28^.

The preforming of the ⌀4.03 m ceramic green body based on gel casting is highly challenging. In fact, the filling procedure requires highly precise control of the rheological properties of the slurry. Whereas the curing process should achieve a complete filling without damaging the preform due to curing stresses. A particular difficulty arises in the drying treatment because drying is a non-stationary diffusion process, and the gradient of the material composition is bound to generate stresses, which are very sensitive to the size of the preform. Thermal stresses resulting from the heat transfer process in the degreasing phase and the significant reduction of the material strength caused by the removal of organic matter, both increase the risk of damaging the large precast green body.

To avoid the aforementioned risks and ensure successful manufacturing, the ⌀4.03 m mirror was structurally split into 12 separate preforms, which were ultimately joined by a proper joining technique. This renders the joining process a critical step in the production chain of the ⌀4 m class SiC mirror blanks.

The existing brazing or diffusion welding technique requires the joint surface to be precisely machined to ensure dimensional accuracy. Nevertheless, the mismatch between the thermal properties of the weld and the base material introduces inevitable additional stresses to the mirror, which restrain the currently used joint techniques to low accuracy applications.

With the superiority of reaction sintering, we used a carbon-based binder to join the degreased green body. The binder is a mixture of SiC powder and phenolic resin. The grain size of the SiC powder is approximately the same as that of the green body. The phenolic resin is used to provide adhesive strength. During reaction sintering, the binder at the weld seam reacts with molten silicon to generate SiC, which is the same material as the body substrate. The joining and reaction sintering were carried out simultaneously to ensure the homogeneous joining of the segmented mirror blanks and eliminating thermal mismatch effects. Additionally, this approach enables a significant reduction in the processing time since the joint surface does not need to be accurately machined.

The workflow adopted here consisted of the following steps. First, the joining layer was optimized to ensure strength under ambient temperature (20–1800 °C) and guarantee safety. Second, the dimensional accuracy and tolerance were relaxed by optimizing the mechanical design. Then, the formula of the joining layer composition was fine adjusted to achieve homogeneous joining. Finally, the joining process was synchronically controlled.

**Fig. 4:**
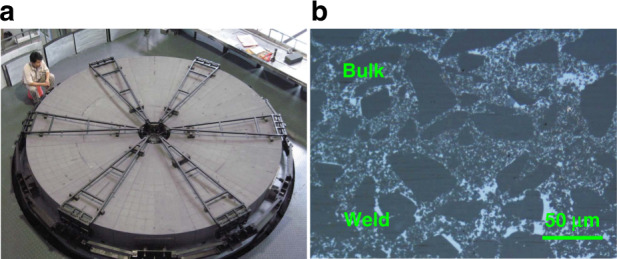
Parts joining and sintering results of ⌀4.03 m SiC blank. **a** Green body parts joining, **b** the microstructure photo after sintering.

**Fig. 5:**
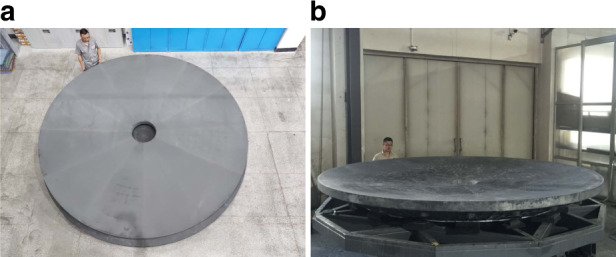
⌀4.03 m SiC blank after parts joining and sintering. **a** Green body, **b** mirror blank after sintering

The material properties are listed in Table 2. Density, elastic modulus, bending strength, fracture toughness, CTE (coefficient of thermal expansion), and thermal conductivity, were tested according to GB standard^29–34^. The joining process and microstructure of the blanks are shown in Fig. 4. The ⌀4.03 m SiC green body after joining, and mirror blank after sintering are shown in Fig. 5.

**Table 2:** Material properties of ⌀4.03 m SiC mirror

Item	Density	Elastic modulus	Bending strength	Fracture toughness	CTE @10–100 °C	Thermal conductivity
Unit	g/cm^3^	GPa	MPa∙m^1/2^	MPa	10^6^/K	W/(m ∙ K)
Value	3.0 ± 0.02	≥350	≥360	≥4.0	2.6 ± 0.2	≥140

### Large SiC aspheric mirror high-accuracy fabrication and cladding technology

The difficulty of fabricating large SiC aspherical mirrors lies in two main aspects. The first consists of meeting the FSF specifications, for instance, 10–15 nm RMS figure error, 5–6 nm RMS mid-spatial frequency (MSF) error, and 1–2 nm RMS roughness. Current techniques are inefficient and unable to control the FSF error. The second aspect is related to the SiC material’s inherent defects (pores, two-phase microstructure, etc.), which should be covered up by the cladding technique. Unfortunately, low stress and high-denseness cladding (or overcoating) technique for ⌀4 m class SiC mirror were not available before 2018.

At CIOMP, we came up with a new processing chain combining the CNC generating, stressed lap grinding/polishing, CCOS and MRF polishing. The new process, together with an efficient converging algorithm, has proven the capability to correct FSF errors effectively. For the MRF polishing machine, the positioning accuracy of each linear and rotary axes is better than 20 μm and 5 arcsec, respectively, as shown in Table 3. Hence, the induced material removal uncertainty is estimated less than 5%. For the ⌀4.03 m SiC aspherical mirror, a 15.2 nm RMS shape error, 6 nm RMS MSF error, and 1 nm RMS roughness have been achieved, whereas the error convergence rate was increased by 30%.

**Table 3:** Axial precision of the 4.5 m MRF polishing machine

Axis #	X	Y	Z	A (rX)
Unit	μm	μm	μm	arcsec
Positioning accuracy	9.3	11.6	3.2	3.7
Repeatability	7.4	9.4	2.4	3.2

A low-temperature Si PVD cladding technique has been developed, where a 10–20 μm-thick Si-cladding layer was deposited on the substrate surface, and the defects on the surface were covered up by Si material. The thickness inhomogeneity of the cladding layer film is less than 5%.

#### Heterocercal stressed lapping

**Fig. 6:**
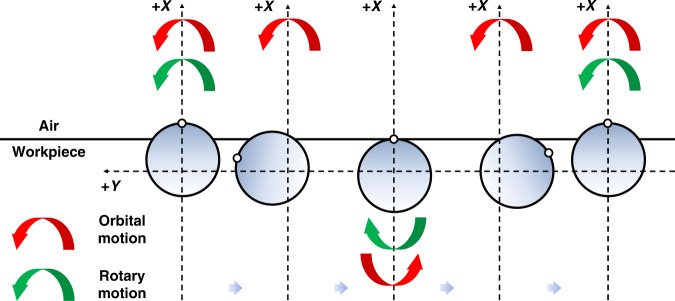
Schematic diagram of heterocercal motion strategy. Double rotary motion is set to enhance the material removal at edge. Red stands for orbital motion direction, while green for spin motion direction

**Fig. 7:**
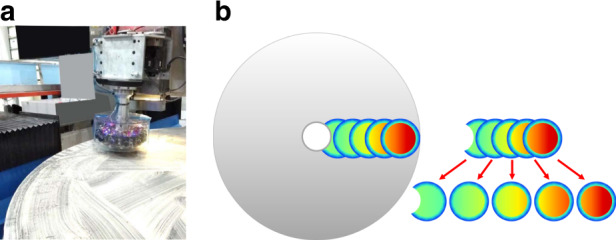
Schematic diagram of the developed large heterocercal stressed lap. **a** ⌀300 mm heterocercal stressed lap device. **b** Typical motion modes and TIFs during process

The stressed lap polishing has proven to be efficient in material removal, particularly in the case of the large aspherical mirror, for the deformable lap can better fit the local curvature of the aspherical surface. However, when the lap moved up to the edge of the mirror, the non-uniform distribution of polishing pressure induces “edge effects”. In a previous study^35^, the heterocercal tool influence function (TIF) has been implemented in a small tool CCOS technique and showed the good capability of controlling the edge, as shown in Fig. 6. We combined the heterocercal TIF with a stressed lap to develop a large heterocercal stressed lap device, as shown in Fig. 7.

The nonsymmetric profile of the heterocercal TIF is intentionally designed to compensate for the non-uniform pressure distribution, and ultimately reducing “edge effect” occurring during the polishing process. This compound motion was implemented based on theoretical simulations and multi-axes motion control hardware. The polishing experimental results of a ⌀3 m off-axis aspherical mirror showed good agreement with computer simulations. Few “rolled edges” or “turned-down edges” were obtained using the heterocercal tool motion.

#### Deterministic fabrication technology for large SiC aspherical mirror

We proposed the concept of convergence time efficiency and defined the criteria for optimal selection of removal function, based on the frequency-domain correlation of surface error^36^.

Based on these new criteria, the tool path and local correction strategy are also optimized. At CIOMP, several deterministic fabrication techniques, such as small tool CCOS, ultra-smooth MRF polishing^37,38^, and ion beam finishing (IBF), have been combined for joint use under the same convergence time efficiency criterion. This compound fabrication strategy has been tried out on a set of large aspherical SiC mirrors (⌀1.5–4 m), demonstrating improved accuracy and efficiency.

The slope-RMS target is included in the merit function for solving the tool dwell time with a deconvolution algorithm, which helps find the global solution.

**Fig. 8:**
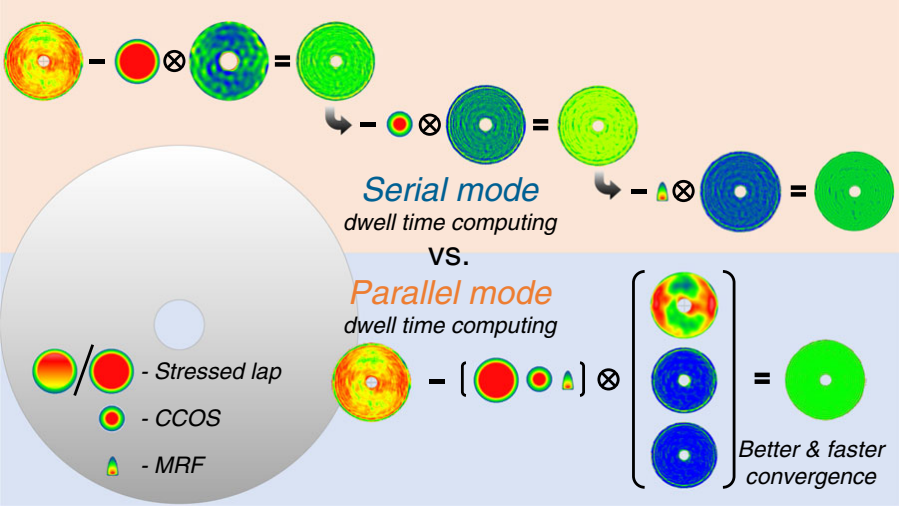
“Parallel calculation and serial run” diagram. Compared to the serial mode, parallel mode shows better and faster convergence in combination fabrication

Figure 8 shows the TIFs of some typical deterministic polishing approaches, such as CCOS, stressed lap, MRF, and IBF. They have different sizes, shapes, and usually different capabilities for correcting the FSF error. In Table 4, we presented the parameters for different tools, such as sizes, volume removal rates (VRR), peak removal rates (PRR), and settings of typical parameters.

**Table 4:** Material removal of sub-aperture tool on PVD Si-cladding RB-SiC substrate

Sub-aperture tool	CCOS	Stressed lap	MRF	IBF
Typical spot				
Spot size/mm	⌀20	⌀340	47.7 × 24.4	FWHM 44.5
VRR (mm^3^/min)	0.105	1.51	0.39	0.71
PRR (μm/min)	0.66	0.02	0.90	0.31
Typical Param. #1	Pad Diam.	Pad Diam.	Wheel Diam.	Grid Diam.
Value	15 mm	300 mm	400 mm	50 mm
Typical Param. #2	Spindle RPM	Spindle RPM	Wheel RPM	Grid Voltage
Value	300	50	60	1003

The conventionally used processing strategy consists in choosing the proper polishing tool according to the measured FSF error and calculating its dwell time function individually. This is called “serial calculation and serial run”. However, in this study, we propose a matrix-based deconvolution model in which the different TIFs are calculated simultaneously. Each dwell time function calculated by the new model is optimized automatically with respect to the FSF error and the correcting ability of the individual tool, which we call the “parallel calculation and serial run”, as shown in Fig. 8. The new calculation model has proven to be more effective compared to the conventionally used one.

#### High-denseness and low-defect cladding technology

**Fig. 9:**
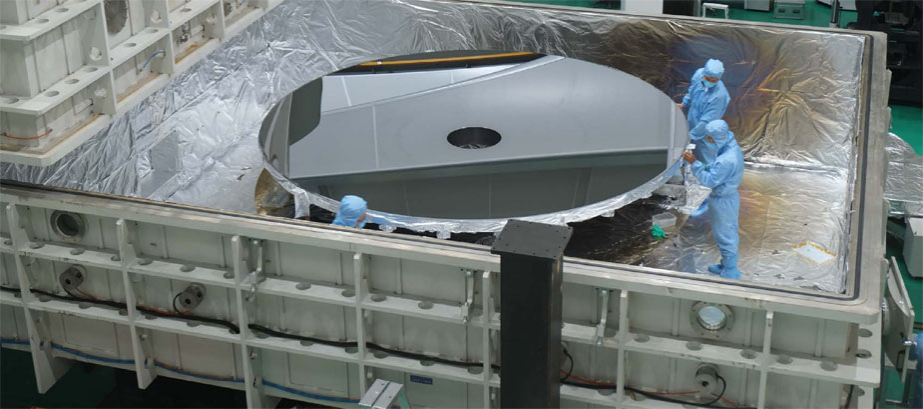
⌀4.03 m SiC mirror after cladding. Its surface is now ready for fine polishing process

**Fig. 10:**
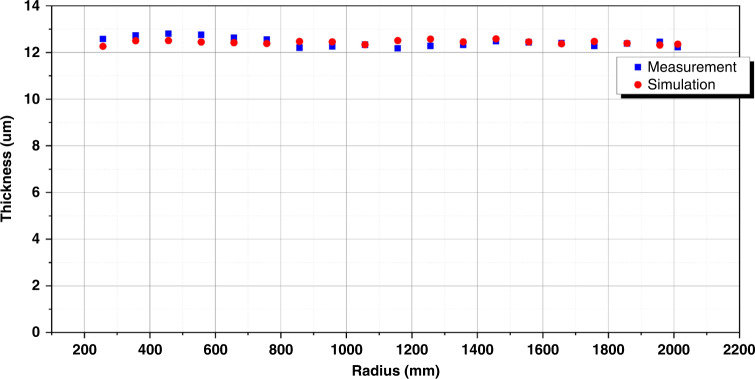
Comparison of the simulated and actual deposition thickness of the cladding layer. The measured thickness inhomogeneity of Si cladding layer is 4.1%

**Fig. 11:**
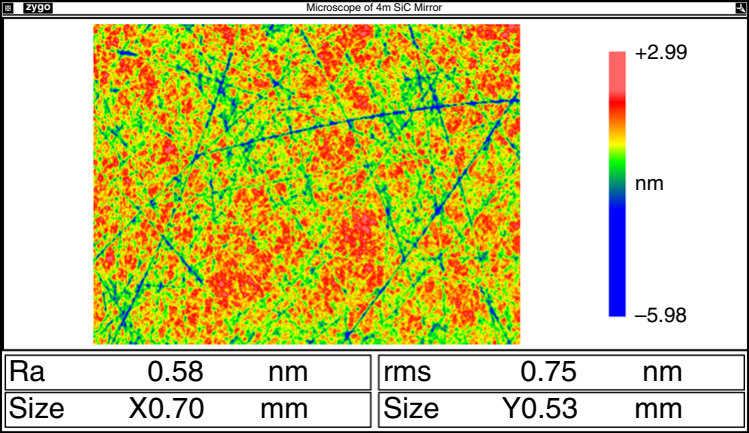
Roughness testing results of the cladding layer after polishing. Surface roughness of the Si cladding layer is polished to 0.8 nm RMS

To eliminate the scattering effect due to the SiC’s inherent defects, we proposed a magnetron sputtering PVD Si-cladding process with a coating temperature lower than 50 °C. A computer-aided large area uniformity adjustment approach was adopted to control the thickness distribution of the cladded Si layer. Thanks to the ion beam clean and sputtering meth, the denseness and adhesion of the cladding layer were greatly enhanced. Figure 9 shows the ⌀4.03 m SiC mirror after PVD SiC cladding. As shown in Fig. 10, the measured thickness of Si-cladding layer agrees well with the simulation data, the inhomogeneity is 4.1%. As shown in Fig. 11, the surface roughness of the Si-cladding layer was polished to 0.8 nm RMS.

### Gravity unload and supporting structure

#### Print-through effect

**Fig. 12:**
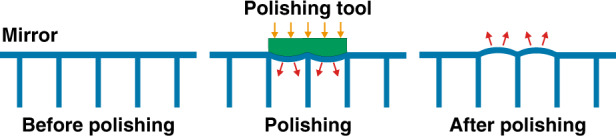
Schematic of the "print-through" effect produced by polishing with large tools. Mirrors with a lightweight structure are subject to “print through” or “quilting” effect under large tool lapping

For ⌀4 m class mirrors, larger polishing tools (≥⌀300 mm) are always selected to produce large material removal and smoothing effects, even though their figuring abilities are inferior to small tools. In practice, large and small tools are alternatively used to balance efficiency and accuracy. Unfortunately, mirrors with a lightweight structure are subject to “print through” or “quilting” effect under large tool lapping, as shown in Fig. 12.

**Fig. 13:**
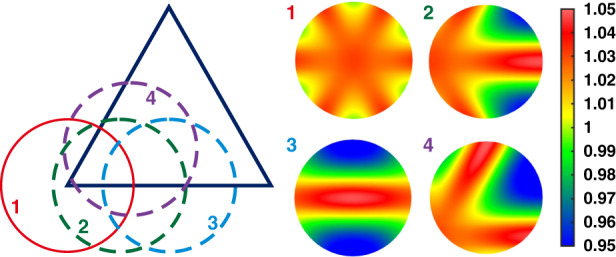
FEM simulation of pressure distribution of polishing tools at different positions. The differences of tool’s pressure distribution at 1–4 points during a triangular lightweight structure polishing. Colorbar stands for the normalized pressure distribution

Quantitative analysis of the “print-through” effect can help optimize the lightweight structure design and the tool size selection as well. The “print-through” effect of various lightweight structures under different polishing tools was quantitatively analyzed by means of the finite element method (FEM)^39^. A prediction model for removal rate was established and used in dwell time calculation to compensate for this effect. Figure 13 shows one example of a triangular lightweight structure under polishing, highlighting the differences in tool’s pressure distribution at 1–4 points.

#### Hydro-unit design

For the primary mirrors in large telescopes, either in space or on the ground, the self-weight-induced deflection must be handled properly. In the case of ground-based telescopes, the gravity-induced deflection shall be quantified for different pitch angles and used as an input to the active compensating unit. Whereas for space telescopes, the gravity-induced deflection must be unloaded during the testing and assembly to ensure their on-orbit performance under a near-zero gravity environment.

To solve the gravity-induced deflection problem, a self-weight unloading support system was developed based on quantitative FEM analysis^40,41^. The deflection caused by gravity was counteracted by the number of force actuators whose distribution was optimized according to the lightweight structure. This technique has been proven valid by several space telescope programs (https://www.cas.cn/syky/201807/t20180723_4659101.shtml)^42^.

**Fig. 14:**
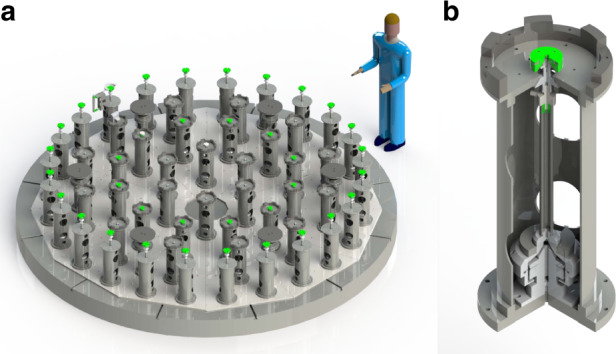
Schematic diagram of the composition principle of the hydrostatic support system and structural design of the hydrostatic unit. **a** Map of Gravity unloading system distribution^40^. **b** Structural design of hydrostatic support module^49^

**Fig. 15:**
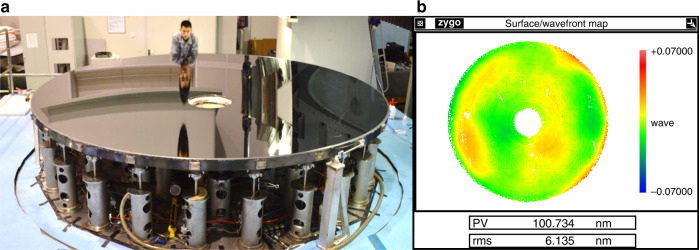
⌀4.03 m SiC mirror gravity unloading system and surface error change after installation. **a** Image of the hydrostatic support system^40^. **b** Changes in the mirror shape after installation

Figure 14 shows the schematic diagram of the self-weight unloading system. On the one hand, the system compensates for the gravity-induced deflection and simulates the near-zero gravity status. On the other hand, it provides an easy-to-operate supporting mechanism to the large mirror under in situ test configuration. The system consists of a hydraulic support module, stiffness adjusting unit, and high-accuracy force feedback control system. Figure 15 shows some details of the hydraulic actuators. For the ⌀4.03 m SiC mirror, the deformation after installation on the assembly is 6.1 nm RMS, which properly meets the specifications.

### Large aspherical optical surface error combination testing technology

Environmental factors, such as mechanical vibrations and air turbulence, directly affect the high-precision test results, particularly for large mirrors with long focal lengths. In addition, to guide the deterministic grinding and polishing, the test instruments must cover the mm to nm range. However, there is no existing single device capable of such a wide dynamic range. For this, we proposed a new approach combining SAP measurement, CGH null interferometry, and phase deflectometry. In addition, an associated data fusion algorithm and a decouple model to subtract air turbulence-induced error from fabrication residuals were established. The test uncertainty for large aspherical mirrors (⌀1–4 m) is better than 6 nm RMS, with a reproducibility better than 2.6 nm RMS.

#### SAP testing technology

**Fig. 16:**
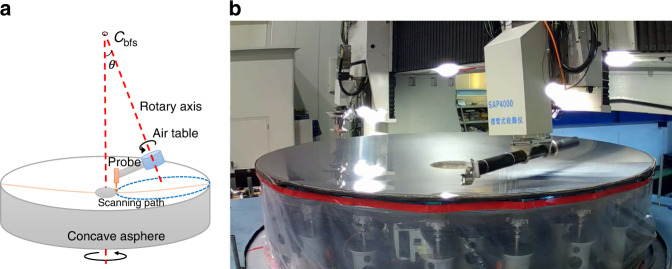
SAP test of the ⌀4.03 m aspherical mirror tested. **a** Schematic diagram of SAP testing method^44^. **b** Built-in SAP testing unit

The SAP was first reported by David Anderson and James Burge^43^ in 1995 (schematic diagram shown in Fig. 16a). In contrast to the traditional sag height measurements, the aspherical departure from the best-fit sphere (BFS) is measured by a short-range probe. Compared to the wieldy used coordinate measuring machine (CMM), the long stroke X, Y, Z linear motions are replaced by two rotary motions, which greatly enhance the robustness and precision^44,45^. For instance, the sag height of the ⌀4.03 m aspherical mirrors is ~170 mm, and departure from BFS is 0.29 mm. Thus, a capacitive displacement sensor, with nano-accuracy, can be used as the test probe. Figure 16b shows a CIOMP-made CCOS machine with a built-in SAP.

**Fig. 17:**
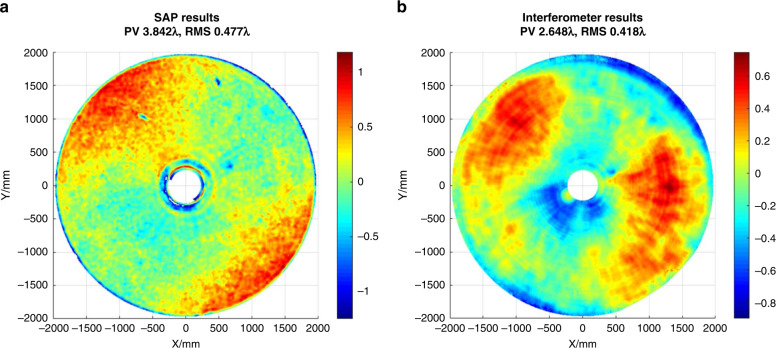
Analysis of the ⌀4.03 m aspherical mirror SAP testing. **a** Test result by SAP. **b** Test result by interferometer

The SAP testing is used to guide the figuring of the ⌀4.03 m mirror from grinding to pre-polishing. For the grinding process, SAP takes about 2.5 h with 72 profiles scanned at each round, with a typical 120 mm lateral resolution. Whereas for the pre-polishing, the SAP takes about 5 h with 144 profiles scanned, and a ~60 mm lateral resolution. As shown in Fig. 17, the SAP test results with 3.84*λ* PV and 0.48*λ* RMS (*λ* = 632.8 nm, hereinafter). This is found in good agreement with the interferometry test result of 2.65*λ* PV and 0.42*λ* RMS. The smaller values obtained from the interferometric test are mainly due to the incapability of the interferometer to resolve the edge errors with high slopes.

#### CGH compensation testing and data fusion technology for large optical complex surfaces

**Fig. 18:**
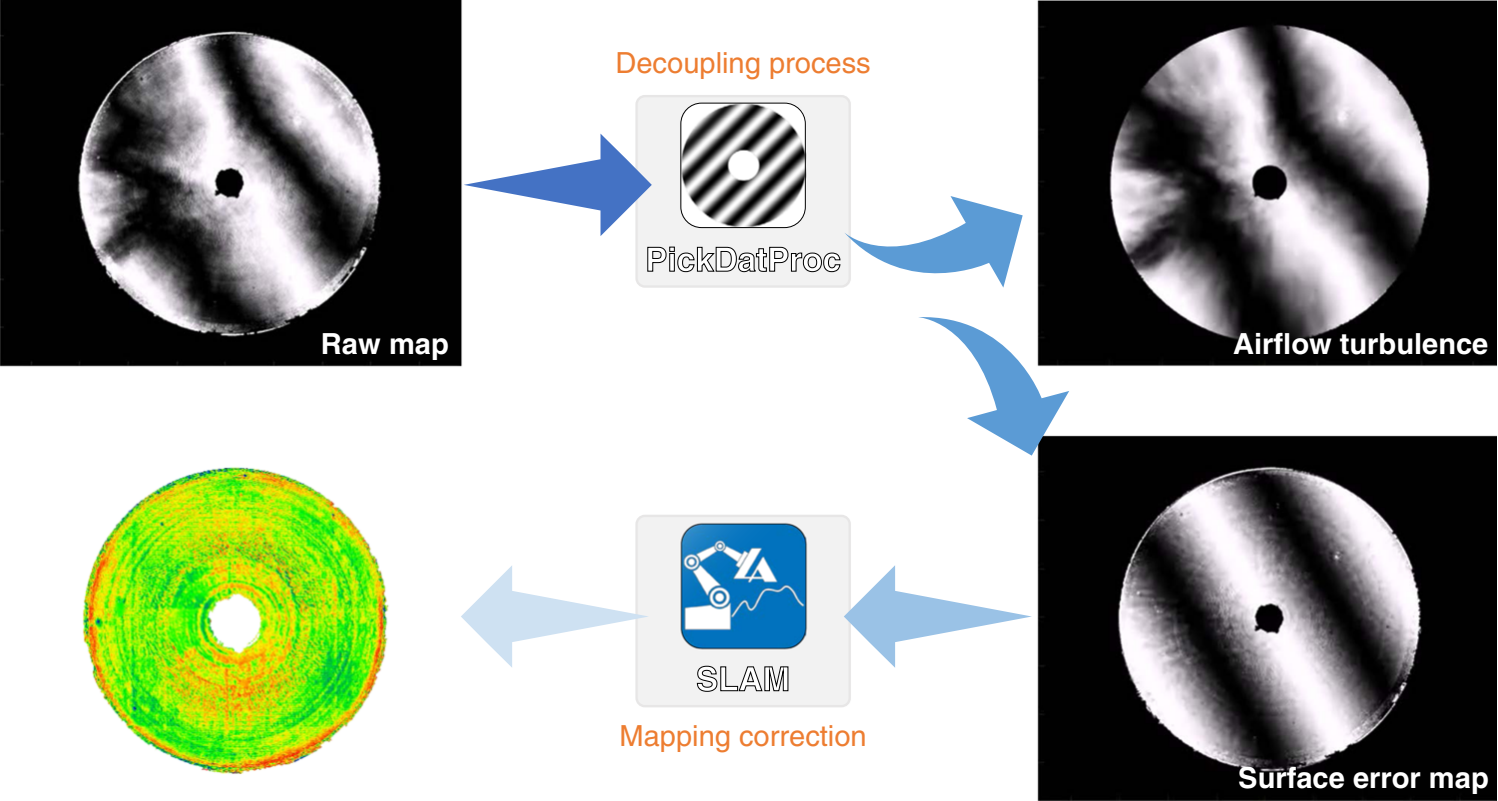
Subtracting the air turbulence induced error during CGH null test. The decoupling algorithm cancels out the effect of air turbulence on large mirror interferometric null test mostly

At CIOMP, we developed the technology of design, fabrication, and calibration of the CGH null test for aspheric or freeform surfaces^46^. In this case, the general uncertainty of the CGH null test is 4.4 nm RMS (*λ*/144), which can be improved to better than 2 nm RMS after calibration. With the decoupling algorithm, the effect of air turbulence on the large mirror interferometric null test was canceled out, as shown in Fig. 18.

**Fig. 19:**
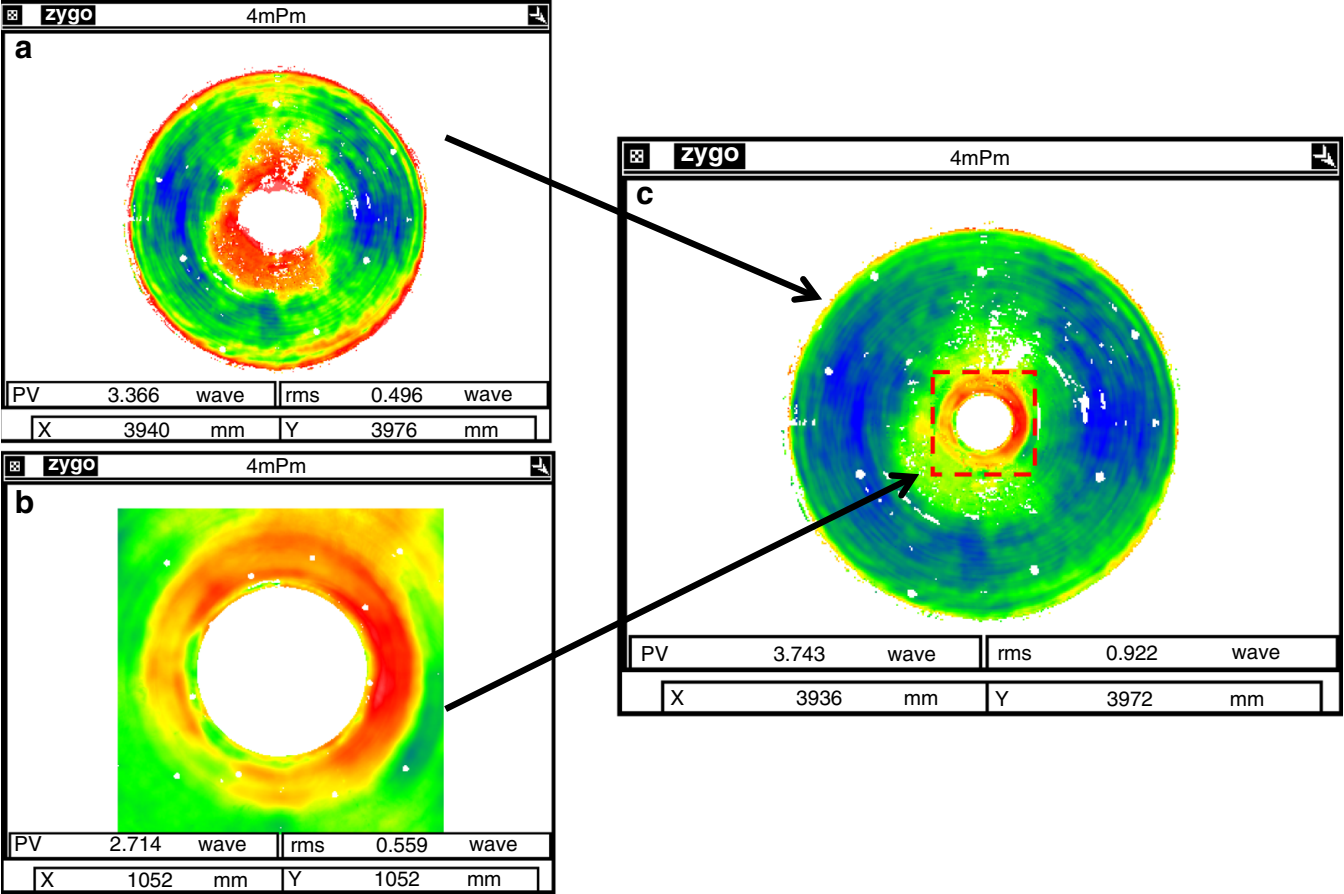
Interferometric test data fusion. **a** CGH null test. **b** data from non-null test, and **c** fused data for the surface of the ⌀4.03 m mirror

In the data fusion process (Fig. 19), CGH interferometry tests the full aperture while the central part is measured directly by the transmission sphere. The non-null test result of the central part contains spherical aberration and will be stored in the computer as a “digital mother plate”.

Based on data fusion techniques, datum from SAP, phase deflectometry, and CGH null interferometry are seamlessly fused to present the FSF errors, with a general test accuracy better than 6 nm RMS and reproducibility better than 2.6 nm RMS.

#### Air turbulence analysis model

When testing the large mirror with a long radius, the effect of air turbulence on test results is significant. Based on the Kolmogorov turbulence theory^47^, a statistical model was established to analyze the area affected by the airflow (Fig. 19). The cone formed by the spherical wavefront is regarded as the integration of the air column with respect to the gradual change of the aperture. Then the power law is extended to the large optical testing condition, and Eq. (1) is derived,

1 $$\Delta _n = {\int}_0^R {{\mathrm{Noll}}\left( n \right)\left( {\frac{D}{R}} \right)^{5/3} \times 0.423k^2C_n^2h^{5/3}dh} = \frac{3}{8}{\mathrm{Noll}}\left( n \right)\left( {\frac{D}{{r_0}}} \right)^{5/3}$$

Here, Δ_*n*_ stands for the mean square error (MSE) of *n*-term standard Zernike residual, Noll(*n*) is the residual coefficient by Noll^48^, *R* and *D* are the height and diameter of the air cone, respectively. They are nearly equal to the radius of curvature and aperture of the mirror under test. *h* is an integral variable along the optical axis; *C*_*n*_ is the structure constant of refractive index introduced by temperature distribution and air turbulence, *r*_0_ is Fried constant or atmospheric coherence length. A set of test results can be fitted statistically according to Eq. (1), and the fitted data helps to find out whether it meets the power law or the homogeneity assumption applies. In addition, the extent of airflow turbulence and coherence length can be directly extracted.

**Fig. 20:**
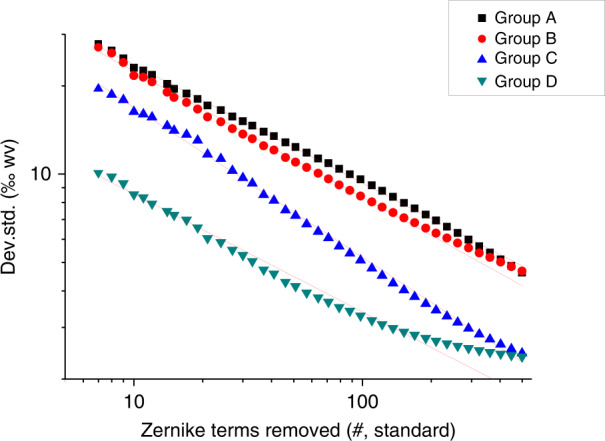
Logarithmic fitting curves of Zernike terms for the effect of air turbulence on interferometric tests. The slope of the curve represents the power relationship in double logarithmic coordinates

Using this method, we analyzed the test results of a ⌀1.45 m mirror with a 10 m optical path. Figure 20 shows the fitted curves of four testing experiments.

In double logarithmic coordinates, the slope of the curve represents its power relationship. Among the four curves shown in Fig. 20, curve C is distinctive and does not follow the power law as the model predicts. The calculation results to quantitatively characterize the airflow environment are listed in Table 5. The fitted parameters also indicate that the Zernike residual error of Group C no longer complies with the equation (Δ_0_ < 0). This implies that the airflow environment in Testing Status C does not meet a random/homogenous turbulence condition, and the result should be regarded as invalid data.

**Table 5:** Calculation datasheet of airflow environment

‰	Δ_0_	*D*_0_/*r*_0_ (better to be small)	*r*_0_ (better to be large)	Adj. R^2^
Group A	7.7 ± 1.1	1.468 ± 0.013	1.0 m	0.99768
Group B	3.4 ± 1.2	1.302 ± 0.014	1.1 m	0.99668
Group C	−6.7 ± 0.8	0.731 ± 0.013	2.0 m	0.99144
Group D	2.01 ± 0.26	0.186 ± 0.003	7.8 m	0.99336

**Fig. 21:**
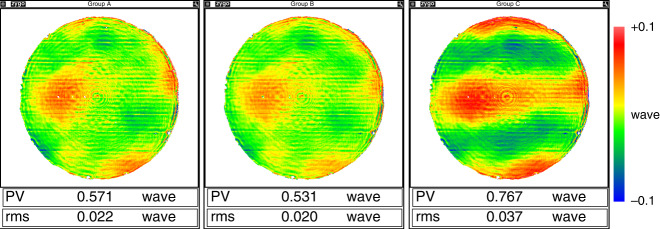
Surface error map tested and comparison. **a** Group A, **b** Group B, **c** Group C

From the experience of the optical specialist, the airflow appears significantly stratified in Group C result, as shown in Fig. 21. Consistently, error map subtraction indicates that Result C is ~50% deviated from others. The proposed method and experience come to the same conclusion.

The analysis model provides a quantitative assessment of the environmental airflow turbulence, and a criterion to judge the test results. This is essential for high-accuracy testing of large aspherical mirrors.

## References

[1] Bauer A, Schiesser EM, Rolland JP (2018). Starting geometry creation and design method for freeform optics. Nat. Commun..

[2] Duerr F, Thienpont H (2021). Freeform imaging systems: fermat’s principle unlocks “first time right” design. Light Sci. Appl..

[3] Hill JM (2010). The large binocular telescope. Appl. Opt..

[4] Xue QS (2019). Spaceborne limb hyperspectral imager for ozone profile detection. Opt. Express.

[5] Wang JH (2019). Tunable liquid lens integrated with aspheric surface. Opt. Commun..

[6] Saunders C (2017). Building large telescopes in orbit using small satellites. Acta Astronautica.

[7] Zhu RZ (2016). Global high-resolution optical satellite overview(2): Europe. Spacecr. Eng..

[8] Hu R (2017). Design optimization method for additive manufacturing of the primary mirror of a large-aperture space telescope. J. Aerosp. Eng..

[9] Comley P (2011). Grinding metre scale mirror segments for the E-ELT ground based telescope. CIRP Ann..

[10] Bos A (2015). Nanometre-accurate form measurement machine for E-ELT M1 segments. Precis. Eng..

[11] Clampin M (2008). The james webb space telescope (JWST). Adv. Space Res..

[12] Postman, M. et al. Advanced technology large-aperture space telescope (ATLAST): A technology roadmap for the next decade. Print at https://arxiv-export1.library.cornell.edu/abs/0904.0941 (2009).

[13] Liu T, Zhou RS (2017). Development overview on GEO high resolution optical imaging system. Spacecr. Eng..

[14] Zhang HD (2017). Modified surface testing method for large convex aspheric surfaces based on diffraction optics. Appl. Opt..

[15] Kirichenko DV, Kleymyonov VV, Novikova EV (2017). Large optical space-based telescopes. Izv.â vys.ših u.čebnyh zavedenij Priborostroenie.

[16] Kim, D. W. et al. Extremely large freeform optics manufacturing and testing. In *Proc. 2015 11th Conference on Lasers and Electro-Optics Pacific Rim*, 1–2 (IEEE, 2015).

[17] Kim, D. W. et al. Advanced technology solar telescope 4.2 m off-axis primary mirror fabrication. In *Proc. Optical Fabrication and Testing 2014*, OTh2B.3 (Optica Publishing Group, 2014).

[18] Webb, K. Advances in fabrication technologies for light weight CVC SiC mirrors. In *Proc. SPIE 6666, Optical Materials and Structures Technologies III*, 666606. (SPIE, 2017).

[19] Piché, F. & Clarkson, A. R. One Year of Finishing Meter-Class Optics with MRF® at L-3 IOS Brashear Optics. In *International Optical Design Conference and Optical Fabrication and Testing, OSA Technical Digest (CD)*, paper OThB6. (Optica Publishing Group, 2010).

[20] Rodolfo, J. et al. SiC mirrors polishing. In *Proc. SPIE 10563, International Conference on Space Optics—ICSO 2014,* 105631Z. (SPIE, 2014).

[21] Pilbratt, G. L. Herschel mission overview and key programmes. In *Proc. SPIE 7010, Space Telescopes and Instrumentation 2008,* 701002. (SPIE, 2008).

[22] Bougoin, M. et al. A new technological step for sic mirrors preparing OTOS. In *Proc. SPIE 10563, International Conference on Space Optics—ICSO 2014*, 105631G. (SPIE, 2017).

[23] Shen F (2007). Influence of RF power on structure and optical properties of polycrystalline silicon thin films. J. Wuhan. Univ. Technol..

[24] Zhang XJ (2019). Development and application of MRF based on robot arm. EPJ Web Conf..

[25] Hu, H. X. et al. Air flow turbulence orthogonality and surface error estimation in large aperture optical testing. In *Proc. SPIE 12166, Seventh Asia Pacific Conference on Optics Manufacture and 2021 International Forum of Young Scientists on Advanced Optical Manufacturing (APCOM and YSAOM 2021)*, 121666B. (SPIE, 2022).

[26] Zhang G (2013). Gelcasting process of 1.5m SiC ceramic green body. Opt. Precis. Eng..

[27] Zhang G (2014). Study on join method of reaction bonded silicon carbide green body. Infrared Laser Eng..

[28] Zhang, G. et al. Fabricating large-scale mirrors using reaction-bonded silicon carbide. *SPIE Newsroom*10.1117/2.1201607.006582 (2016).

[29] Test methods for density and apparent porosity of fine ceramics, (GB/T 25995-2010), Standards press of China.

[30] Test methods for elastic moduli of fine ceramics (advanced ceramics, advanced technical ceramics)- Bending method, (GB/T 10700-2006), Standards press of China.

[31] Fine ceramics (advanced ceramics, advanced technical ceramics)—Test method for for flexural strength of monolithic ceramics at room temperature, (GB/T 6569-2006), Standards press of China.

[32] Fine ceramics (advanced ceramics advanced technical ceramics)—Test method for fracture toughness of monolithic ceramics at room temperature by single edge precracked beam (SEPB) method, (GB/T 23806-2009), Standards press of China.

[33] Fine ceramics (advanced ceramics advanced technical ceramics)—Test method for linear thermal expansion of monolithic ceramics by push-rod technique, (GB/T 16535-2008), Standards press of China.

[34] Determination of thermal diffusivity or thermal conductivity by the flash method, (GB/T 22588-2008), Standards press of China.

[35] Hu HX (2016). Edge control in a computer controlled optical surfacing process using a heterocercal tool influence function. Opt. Express.

[36] Hu, H. X. *Research on the key technologies in the application of space optical freeform surfaces*. PhD thesis, Changchun Institute of Optics, Fine Mehcanics and Physics, Chinese Academy of Sciences, Changchun, (2017).

[37] Bai Y (2016). Rapid fabrication of a silicon modification layer on silicon carbide substrate. Appl. Opt..

[38] Bai Y (2022). Material removal model of magnetorheological finishing based on dense granular flow theory. Light Adv. Manuf..

[39] Qiao GB (2021). Stress-induced deformation of the coating on large lightweight freeform optics. Opt. Express.

[40] Hu HF (2019). Designing a hydraulic support system for large monolithic mirror’s precise in-situ testing-polishing iteration. Opt. Express.

[41] Hu HF (2017). Hydrostatic support system for in-situ optical testing of a 4 m aperture SiC mirror. Opt. Precis. Eng..

[42] Changchun Institute of Optics, Precision Machinery and Physics, Chinese Academy of Sciences. (2018). Manufacturing facilities system for 4m-class high precision SiC aspheric mirrors. Bull. Chin. Acad. Sci..

[43] Anderson, D. S. & Burge, J. H. Swing-arm profilometry of aspherics. In *Proc. SPIE 2536, Optical Manufacturing and Testing*. (SPIE, 1995).

[44] Xiong L (2019). Stitching swing arm profilometer test for large aperture aspherics. Chin. Opt. Lett..

[45] Xiong L (2016). Swing arm profilometer: analytical solutions of misalignment errors for testing axisymmetric optics. Optical Eng..

[46] Zhang, X. J. et al. Manufacturing and testing large SiC mirrors in an efficient way. In *Proc. SPIE 9628, Optical Systems Design 2015: Optical Fabrication, Testing, and Metrology V*, 96280S. (SPIE, 2015).

[47] Birnir, B. *The Kolmogorov-Obukhov Theory of Turbulence: A Mathematical Theory of Turbulence*. (Springer, 2013).

[48] Noll RJ (1976). Zernike polynomials and atmospheric turbulence. J. Optical Soc. Am..

[49] Hu, H. F. et al. *Static pressure supporting device for ultra large diameter optical machining*. Chinese patent, ZL201310339253.7, (2013).

